# Hospital-Based Study of Epithelial Malignancies of Endometrial Cancer Frequency in Lahore, Pakistan, and Common Diagnostic Pitfalls

**DOI:** 10.1155/2014/179384

**Published:** 2014-01-06

**Authors:** Imrana Tanvir, Sabiha Riaz, Afshan Hussain, Riffat Mehboob, M. Usman Shams, Haseeb Ahmad Khan

**Affiliations:** ^1^Fatima Memorial College of Medicine and Dentistry, Lahore 54000, Pakistan; ^2^Department of Biomedical Sciences, King Edward Medical University, Lahore 54000, Pakistan

## Abstract

The current study was conducted to see the frequency of epithelial malignancies of endometrium with focus on the common diagnostic pitfalls and identify morphological and immunohistochemical markers helpful in the differential diagnosis between different subtypes. It is a retrospective descriptive study carried out on 52 specimens of endometrial tumors received in Fatima Memorial Hospital, Lahore, Pakistan, during three years (2010–2012). Patients were divided into 5 age groups: <40, 41–50, 51–60, 61–70, and >70 yrs. Tissues were fixed in 10% formalin and processed and stained with haematoxylin-eosin. Stained slides were examined to determine the histological types by WHO classification, and immunohistochemistry for WT1, p53, ER/PR, and MIB1 was done in cases where morphology alone was not helpful in making a confirmed diagnosis. 80% of specimens were of endometrioid adenocarcinomas, 11% of serous tumors, 4% of clear cell carcinoma, and 4% of squamous cell carcinomas involving both cervix and endometrium. Most of the patients (28.84%) with endometrial carcinomas fall in the age range of 51–60 yrs. Endometrioid adenocarcinoma is the most common type of epithelial endometrial malignancies. Morphology is the keystone in the evaluation of these tumors, but immunohistochemistry can also be helpful in establishing the correct diagnosis.

## 1. Introduction

Endometrial cancer is the most common gynecologic malignancy in western women with 41,000 new cases projected in the United States for 2006 [1], whereas rates in developing countries and Japan are four to five times lower. In India, the rates are as low as 4.3 per 100,000 [2]. Ninety-seven percent of all cancers of the uterus arise from the glands of the endometrium and are known as endometrial carcinomas [3]. Its annual incidence is estimated at 10–20 per 100,000 women and it is increasing [4, 5]. Approximately, 75% of cases are diagnosed at an early stage with a tumor confined to the uterine corpus [6]. It is the fourth most common cancer in women after carcinomas of breast, colorectum, and lung [7]. In the United States, endometrial carcinoma accounts for approximately 6000 deaths per year [4].

The median age of patients at the diagnosis of endometrial carcinoma is 63 years [8]. The incidence of endometrial carcinoma is highly dependent on age; there are 12 cases per 100,000 women at 40 years of age and 84 per 100,000 at 60 years [3]. Five percent of women with endometrial cancer are less than 40 years of age. Seventy-five percent of women with endometrial carcinoma are postmenopausal [9].

Historical observations have suggested that endometrial carcinomas vary in histopathologic appearance and clinical features [10]. It is composed of a number of tumor types with different light-microscopic features, molecular genetic alterations, and prognoses [11]. The microscopic appearance of the tumor resembles that of the proliferative endometrium, with a variable degree of glandular complexity and cellular pleomorphism [12].

In 1983, Bokhman [13] first proposed the hypothesis of two distinctly different forms of endometrial carcinoma and their associated differences in risk factors and prognosis based on light microscopic appearance, clinical behavior, and epidemiology. Type 1 is estrogen-related endometrioid carcinoma divided into subtypes, adenocarcinomas with squamous differentiation further subdivided into adenocarcinoma with squamous metaplasia (adenoacanthoma) and adenosquamous carcinoma, secretory, ciliated cell, and villoglandular variants [14]. Type 2 is nonestrogen-related, nonendometrioid carcinoma and includes uterine papillary serous carcinoma (UPSC), clear cell carcinoma (CC), and mucinous and squamous cell carcinoma [7]. Recently recognized subtypes are the tumors that arise in the setting of hereditary nonpolyposis colon cancer syndrome tumors with small nonvillous papillae, presence of microglandular pattern, sertoliform features, and dedifferentiated carcinomas [12].

Studies suggest that the most common type of endometrial carcinoma is endometrioid adenocarcinoma, which is composed of malignant glandular epithelial elements with an admixture of squamous metaplasia [15]. It develops from endometrial hyperplasia in the setting of excess estrogen exposure [10].

Clear cell and papillary serous carcinoma of the endometrium are tumors that are histologically similar to those noted in the ovary and the fallopian tube, and the prognosis is worse for these tumors [16]. Mucinous, squamous, and undifferentiated tumors are rarely encountered. Endometrioid accounts for 75%–80%, uterine papillary serous (<10%), clear cell (4%), and squamous cell (<1%), and mixed endometrial carcinoma is 10%. Serous carcinomas develop from “endometrial intraepithelial carcinoma,” a lesion representing malignant transformation of the endometrial surface epithelium [10].

We in this study tried to document our experiences regarding different types of epithelial malignancies in endometrium. We also stressed on classic morphologic feature that remains the key in diagnosis; however, in difficult cases immunohistochemistry is quite helpful in establishing correct diagnosis [17, 18].

## 2. Materials and Methods

It is a hospital-based retrospective study on women of all ages divided into 5 groups. Authors collected 52 biopsy specimens of patients during three years (2010–2012) referred to Fatima Memorial Hospital, Lahore, Pakistan. This study was in accordance with the Declaration of Helsinki. Approval for the study was taken from the review board of the ethical committee of the hospital [19]. All the endometrial biopsies and the hysterectomies specimens received in the last 3 years were retrieved from hospital records and evaluated. Slides were stained with hematoxylin Eosin and only the confirmed cases for epithelial malignancies of endometrium diagnosed by two qualified histopathologists were included in the study (Table 1). Immunohistochemistry for WT1, p53, estrogen/progesterone (ER/PR), and MIB1 was performed on these cases. Immunohistochemical staining was performed on formalin-fixed, paraffin-embedded 4-*μ*m sections. The tissue sections were deparaffinized and incubated in 1% hydrogen peroxidase for 10 minutes to quench endogenous tissue peroxidase. Antigen retrieval was carried out in citric buffer in microwave (high power) for 10 min. The tissue sections were then incubated with the antibodies. The slides were stained using a standard EnVision kit (DAKO) according to the manufacturer's protocol. Immunohistochemical reactions were developed with diaminobenzidine as the chromogenic peroxidase substrate, and slides were counterstained with hematoxylin. Negative control samples were included. Simple percentages of the number of cases of endometrial cancers according to type and ages were calculated.

## 3. Results

In this study, 52 confirmed cases of epithelial malignancies were examined to determine the most frequent type of epithelial endometrial cancer and its association with age. Out of 52 cases, 42/52 (80%) had endometrioid carcinoma, 6/52 (11%) had serous carcinoma, 2/52 (4%) had clear cell carcinoma and 2/52 (4%) had squamous cell carcinoma (Table 1). Maximum patients belonged to age group of 51–60 years (Table 4).

## 4. Discussion

Ability to diagnose adenocarcinoma in an endometrial sampling is highly dependent on adequacy of the specimen. The endometrial sampling is a screening tool, but unfortunately not all of the endometrium may be represented in any given sample, so the presence of a myometrial lesion cannot be assessed. Most pathologists and surgeons assume that the presence of cancer in the myometrium (myoinvasive cancer) is associated with cancer in the endometrium. In all such instances where the biopsy specimen is inadequate, resampling with additional imaging studies should be considered, especially if there is a concern for adenocarcinoma. At our institution, we receive all kinds of specimens (Table 2). The endometrial-curettage specimen can provide important information regarding the histologic type and grade of the tumor [3]. We encountered malignancies in different age groups (Table 3). Advanced age adversely affects survival in endometrial carcinoma. Women with papillary serous, clear-cell, or adenocarcinoma with squamous differentiation have an older median age than women with endometrioid adenocarcinoma [8].

Christopherson et al. described adenocarcinoma, adenoacanthoma, adenosquamous carcinoma, clear-cell carcinoma, and papillary serous carcinoma in 60%, 22%, 7%, 6%, and 5% of the cases [22]. The differential diagnosis of endometrial hyperplasia and well-differentiated endometrioid adenocarcinoma is complicated not only by the resemblance of these lesions to each other, but also by their tendency to be overdiagnosed (particularly hyperplasia) on the background of polyps, endometritis, artifacts, and even normally cycling endometrium. Low-grade adenocarcinomas are best recognized by architectural evidence of stromal invasion, usually in the form of stromal disappearance, desmoplasia, necrosis, or combinations of these findings between adjacent glands. Endometrioid adenocarcinomas are usually Type 1 cancers associated with manifestations of endogenous or exogenous hyperestrogenic stimulation and a favorable prognosis [14].

Morphology is the key to the diagnosis and subtyping of these biopsies; however, this should be combined with clinical history, gross evaluation, and appropriate sampling. Classical morphological features usually allow for correct diagnosis. Difficulties may arise when tumor show unusual morphology, are, high grade, or mixed. Nonprimary endometrial carcinoma for example, tumors of cervix, fallopian tube, ovary, peritoneum, or other pelvic organs can also mimic different subtypes of endometrial tumors and can be of diagnostic challenges.

The most extensively studied biologic markers in endometrial carcinoma are estrogen and progesterone receptors (Table 5) [3]. Immunohistochemistry can also be helpful, if interpreted in the right context in reaching a correct diagnosis. In the recent era, pathologists are trying to study role of different immunomarkers and their value as diagnostic tool in endometrial cancers. The following markers are being studied for their potential value in differential diagnosis of P16, WT1, PTEN, PAX2, P53, mammaglobin, and so forth are being studied for their potential value in differential diagnosis.

**Figure 1:**
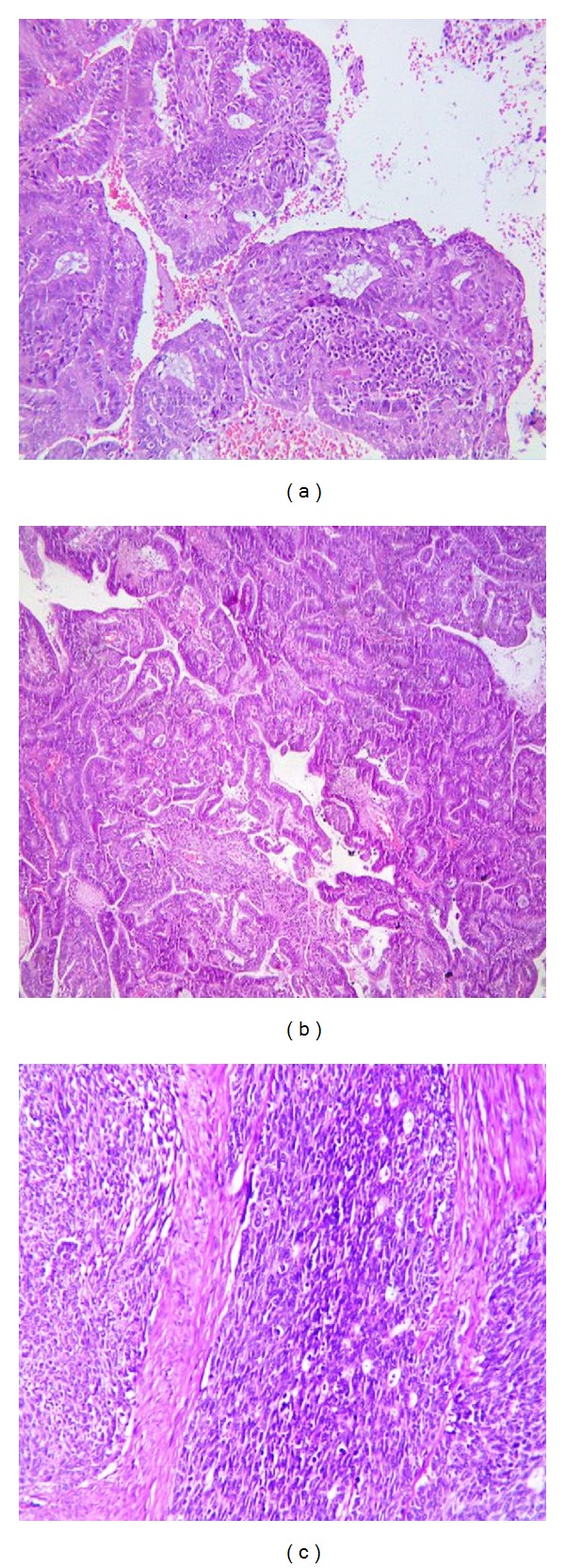
Hematoxylin Eosin stained sections of endometrial carcinoma ((a), (b)) endometrioid adenocarcinoma, (c) poorly differentiated adenocarcinoma.

**Figure 2:**
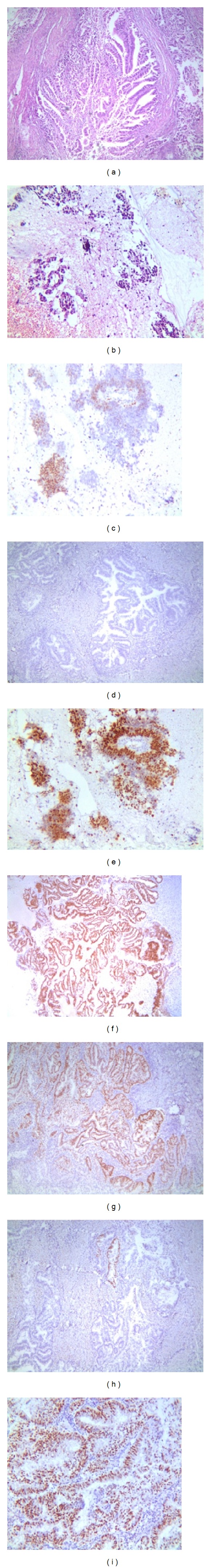
(a) H&E stained section of endometrioid carcinoma (b) H&E stained section of serous carcinoma (c) positive staining for WT-1 in Serous Carcinoma (d) Negative staining for p53 in Serous carcinoma (e) Positive staining for P53 in Serous Carcinoma ((f) and (g)) Positive staining for ER in Endometrioid Carcinoma (h) PR negative (i) Positive staining for PR in Endometrioid Carcinoma.

In most of the instances, it is simple to diagnose different subtypes of endometrial carcinomas based on their characteristic morphology. We at our institution observed the same experience and morphology is the key factor in diagnosing endometrial carcinomas (Figures 1 and 2) (Table 1). When endometrioid adenocarcinoma is well to moderately differentiated, it closely resembles normal endometrium showing architectural complexity, cribriforming, and overcrowding of the glands (Figure 1(b)) [12, 23, 24]. In contrast, serous carcinomas typically exhibit irregular, branching papillae with budding small papilla. The neoplastic cells show large pleomorphic nuclei (Figure 2(b)) [23–25]. At times, it is difficult to make a correct diagnosis if predominant histologic pattern deviates from normal morphology [17].

Endometrial serous carcinoma with predominant glandular component can be mistaken for endometrioid adenocarcinoma [26]. Most important clue to diagnosis is extreme discordance of architectural and cytological features. Other important features are lack of polarity pseudostratification, striking pleomorphism, brisk mitosis, and single cell apoptosis, feature which are absent in endometrioid adenocarcinomas [26]. If adequately sampled typical morphology is usually encountered. In 4 of our case we were unable to diagnose on morphology alone. In one case the sample was limited and morphology was not quite helpful. Those difficult cases were of serous carcinomas and were poorly differentiated and also showed some glandular pattern, but as the cytological atypia was high. In these cases, we used immunohistochemical markers P53, WT1, ER, and PR (Table 5). P53 showed strong diffuse staining which is typically associated with serous carcinoma and a high MIB1 index [27, 28] (Table 5).

Lab-based analyses beyond the usual diagnosis based on light microscopic examination of H&E stained slides—immunohistochemistry and PCR-based assays such as sequencing, mutation testing, microsatellite instability analysis, and determination of MLH1 methylation—are most helpful for guiding diagnosis and treatment of endometrial cancer [29].

## 5. Conclusion

Our approach to the diagnosis of well-differentiated endometrial adenocarcinoma in biopsy, curettage, and hysterectomies, is based primarily on glandular architecture and cytological features. Adequate sampling with thorough morphologic assessment and immunoprofile is essential for accurate assessment. Morphology is the key when subcategorizing tumors; however, in high grade tumors immunohistochemistry is of valuable help.

## Figures and Tables

**Table 1:** Frequency of different types of endometrial cancers.

Type of cancer	Cases	Percentage
Endometrioid carcinoma	42	80%
Serous carcinoma	6	11%
Clear cell carcinoma	3	5%
Squamous cell carcinoma	2	4%

Total	52	100%

**Table 2:** Comparison of frequency of endometrial carcinoma in this study with previously reported studies.

Endometrioid Carcinoma (%)	Nonendometrioid Carcinoma (%)	References
80	20	This study
87	13	[20]
87	13	[21]
89	11	[3]

**Table 3:** Type of specimens.

Type of specimen	Cases	Percentage
Endometrial curretings	18	34.6
TAH and BSO	15	28.8
TAH	04	7.6
Pipelle	07	13.4

**Table 4:** Age range of patients with endometrial carcinomas.

Age range	Cases	Percentage
Below 40 yrs	03	5.7
41–50	10	19.2
51–60	15	28.8
61–70	10	19.2
Above 70	06	11.5

**Table 5:** Immunomarkers in uterine serous verses uterine endometrioid carcinoma.

	WT1	P53	ER/PR	MIB1
Serous	+/−	+	−/+	High
Endometrioid	−	−/+	+/−	low
